# A three-dimensional discrete element model of triaxial tests based on a new flexible membrane boundary

**DOI:** 10.1038/s41598-021-84224-7

**Published:** 2021-02-26

**Authors:** Yan Qin, Chun Liu, Xiaoyu Zhang, Xingang Wang, Bin Shi, Yue Wang, Shang Deng

**Affiliations:** 1 School of Earth Sciences and Engineering, Nanjing University, Nanjing, 210023 China; 2 Nanjing University (Suzhou) High-Tech Institute, Suzhou, 215123 China; 3 Department of Geology, Northwest University, Xi’an, 710127 China; 4 Petroleum Exploration and Production Research Institute, SINOPEC, Beijing, 100083 China

**Keywords:** Solid Earth sciences, Engineering

## Abstract

Based on a new elastic clump model, a flexible membrane is proposed for the discrete element numerical simulations of triaxial tests. Conversional triaxial tests of sandstone under the confining pressures of 2 MPa and 8 MPa were carried out, in order to validate the effectiveness of the proposed numerical simulation method. The numerical model is validated by comparing the numerical results with the test results. The deformation and failure process of numerical model is analyzed by stress–strain curves, micro fractures, displacement fields, stress fields and energy fields. The model shows an X-shape shear failure zone, of which the angle is very close to that of the test; the dip angle of most shear fractures is close to the angle of the internal friction; and there is a large amount of slipping frictional heat generated on the failure surface. During the loading process, the stress chain and stress concentration appear in the middle of the model, which lead to displacement zoning in the model. The failure of the model is associated with the growth of the micro tensile- and shear fractures. This study provides an effective tool for the macro–micro investigation of rock failure processes.

## Introduction

The conventional triaxial test is one of the standardized laboratory tests, which plays a fundamental role in geotechnical engineering researches^1^. By using the equipment, the stress–strain curve of a rock block under special confining pressure can be obtained, so as to investigate the deformation and failure process of the rock under complicated conditions. Combined with microscopic analyses, we are able to analyze and understand the micro mechanisms of the failure processes and patterns of rock and soil mass. However, comprehensive experiments and micro analyses are time-consuming and challenge, computer numerical simulations provide a very important way to decipher the macro–micro relationship of rock and soil mass^2,3^.

The discrete element method (DEM) has been demonstrated as a powerful numerical tool since Cundall and Strack introduced it to the geotechnical engineering community to study the behavior of granular material^4^. The complex disintegration of the particulate system, such as rock and soil, can be well simulated by using the DEM^5,6^. Therefore, the method has been used to simulate triaxial tests^7–10^, direct shear test^11,12^ and landslide^13,14^, etc.

In the DEM numerical simulation of triaxial tests, the cylindrical soil or rock specimens generally are enclosed in an elastic latex membrane to isolate it from the surrounding confining medium^15^. Rigid plates are attached to the top and bottom sides of the specimen. Apart from the generation of the specimen, the boundary condition will have a large influence on the simulation results, especially the lateral boundary. Usually, three kinds of lateral boundaries are adopted in the numerical simulation of triaxial tests, the periodic, rigid and flexible boundaries. A periodic boundary assumes that the specimen is surrounded by identical elements in all directions, this boundary is commonly used in the simulation of parallelepiped specimens, such as the true triaxial test^16–18^. However, its application in the conventional triaxial test is difficult, because a periodic boundary always has an exact copy plane on the opposite side, it’s hard to find an opposite plane for cylindrical boundary accurately^19^. The rigid boundary is the most widely used type of boundary, in combination with a servo controlled system to control the confining pressure, a cylindrical rigid wall can control the lateral boundary condition effectively by moving these boundaries slowly inwards or outwards^17,20,21^. The drawbacks of rigid boundaries are that the boundary elements tend to inhibit the failure process and deformation of the specimen^22^.

The flexible boundary can be divided into two different types. The first one presented in 1990s, it ignores the existence of membrane and directly applies the pressure generated by the membrane to the outermost elements equivalently^16,23^. Because no boundary elements are used in this approach, the membrane-specimen interaction could not be examined^15^. The other strategy using a flexible membrane that composed of a series of bonded elements. 2D biaxial models are generally used because of the large amount of calculation of 3D model^24^. Further, Cil and Alshibli proposed a flexible membrane boundary model consisting of spherical elements in PFC^3D^,the hexagonal element arrangements were adopted for the flexible membrane; the flexible membrane can replicate the confining stress uniformly applied in real triaxial tests and predict the macroscopic stress–strain behavior and deformation characteristics of granular materials, however, small elements may leak out of the gap between the membrane elements, in particular, when under large compressive strains^15^. To address the problem, the membrane elements must be small enough, which will significantly increase the number of elements and decrease the speed of numerical simulations.

In this paper, a three-dimensional discrete element model based on a flexible membrane boundary is built to simulate the conversional triaxial tests. The membrane is made up of a deformable elastic clump, there is no gap and hole on the membrane surface, so it is relatively smooth and can effectively prevent elements from leaking out. Subsequently the confining pressure was decomposed to each membrane element. Triaxial tests of sandstone under different confining pressures were carried out^25^, and the numerical simulations corresponding to the triaxial tests were carried out, too. In the numerical tests, the ideal stress–strain curves were obtained and the shear failure of the numerical model were observed. Finally, the micro mechanism of model failure was analyzed.

## The discrete element method

### The contact model of elements

**Figure 1:**
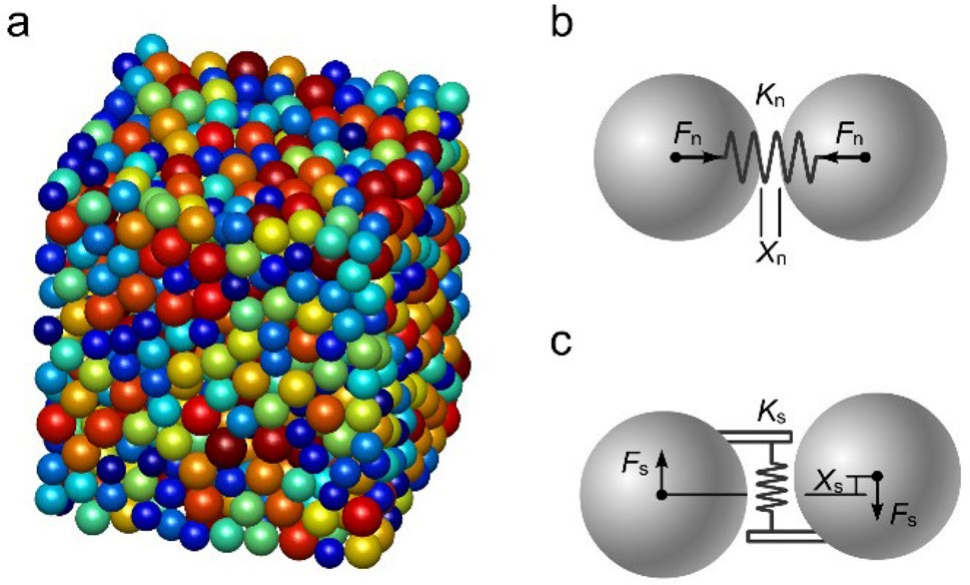
(**a**) A 3D discrete element model; (**b**) Normal spring force; (**c**) Shear spring force.

As shown in Fig. 1a, in the DEM, rock and soil are represented by an assemblage of a series of bonded discrete elements, which have specific mechanical properties. In the linear elastic contact model, it is assumed that elements interact with each other via spring forces. The normal force (*F*_n_) and normal deformation (*X*_n_) between two elements can be simulated by a normal spring^26,27^:

1 $$F_{\text{n}} = \left\{ {\begin{array}{*{20}l} {K_{\text{n}}X_{\text{n}},X_{\text{n}} < X_{\text{b}}} \hfill & {\text{intact bond}} \hfill & {\text{(a)}} \hfill \\ {K_{\text{n}}X_{\text{n}},X_{\text{n}} < {0}} \hfill & {\text{broken bond}} \hfill & {\text{(b)}} \hfill \\ {{0},X_{\text{n}} \ge {0}} \hfill & {\text{broken bond}} \hfill & {\text{(c)}} \hfill \\ \end{array} } \right.$$

where *K*_n_ is the normal stiffness; *X*_n_ is the normal displacement relative to the equilibrium position (Fig. 1b); *X*_b_ is the breaking displacement, which is the relative displacement of two elements with the maximum normal tensile force. Note that, *X*_n_ is defined as the displacement relative to the equilibrium position (*X*_n_ = 0), but not the overlap of elements as many previous studies^28^. It is also used in the calculation of the force between the clump elements in the next section, so as to avoid confusion with the “overlap”. The elements are originally connected with adjacent elements and subjected to tensile (positive) or compressive forces (Eq. 1a). When the normal relative displacement is greater than the breaking displacement between two elements, the normal spring is broken and the tensile force is disappeared. In case of a broken bond (Eqs. 1b-c), the inter-element normal force is always zero when the relative displacement is greater or equals to zero (Eq. 1c), while the repulsive force still acts when they return to a compressive contact (Eq. 1b).

Similarly, the shear force (*F*_s_) and shear deformation (*X*_s_) between two elements are simulated by a tangential spring^4,29^:

2 $$F_{{\text{s}}} = K_{{\text{s}}} X_{{\text{s}}}$$

where *K*_s_ is the shear stiffness; *X*_s_ is the shear relative displacement (Fig. 1c). The failure of the spring in tangential direction is based on the Mohr–Coulomb criterion:

3 $$F_{{{\text{S}}\max }} = F_{{{\text{s}}0}} - \mu_{{\text{p}}} F_{{\text{n}}}$$

where *F*_s max_ is the maximum shear force; *F*_s0_ is the inter-element initial shear force; *μ*_p_ is the inter-element coefficient of friction. In the Mohr–Coulomb criterion, the maximum shear force between elements is related to the initial shear force. When the normal force (*F*_n_) is zero, *F*_s0_ is the maximum shear force, which is similar to the cohesion of rock and soil mass. The shear force increases with the increasing of normal force (*F*_n_). When the tangential force exceeds the maximum shear force, the tangential spring is broken, and two elements begin slipping, the slipping friction force is − *μ*_p_·*F*_n_*.*

When the force of each element is obtained, the displacement of the element can be calculated based on the Newton equation of motion and a time step iteration algorithm^4^. In a very small step time, the motion of the element is regarded as linear, and the force, acceleration, velocity and displacement of the element can be calculated. Once the current time step calculation is completed, a time step is advanced to implement a new iteration of the DEM. Generally, millions to tens of millions of iterations are required in a DEM numerical simulation.

### The elastic clump model

**Figure 2:**
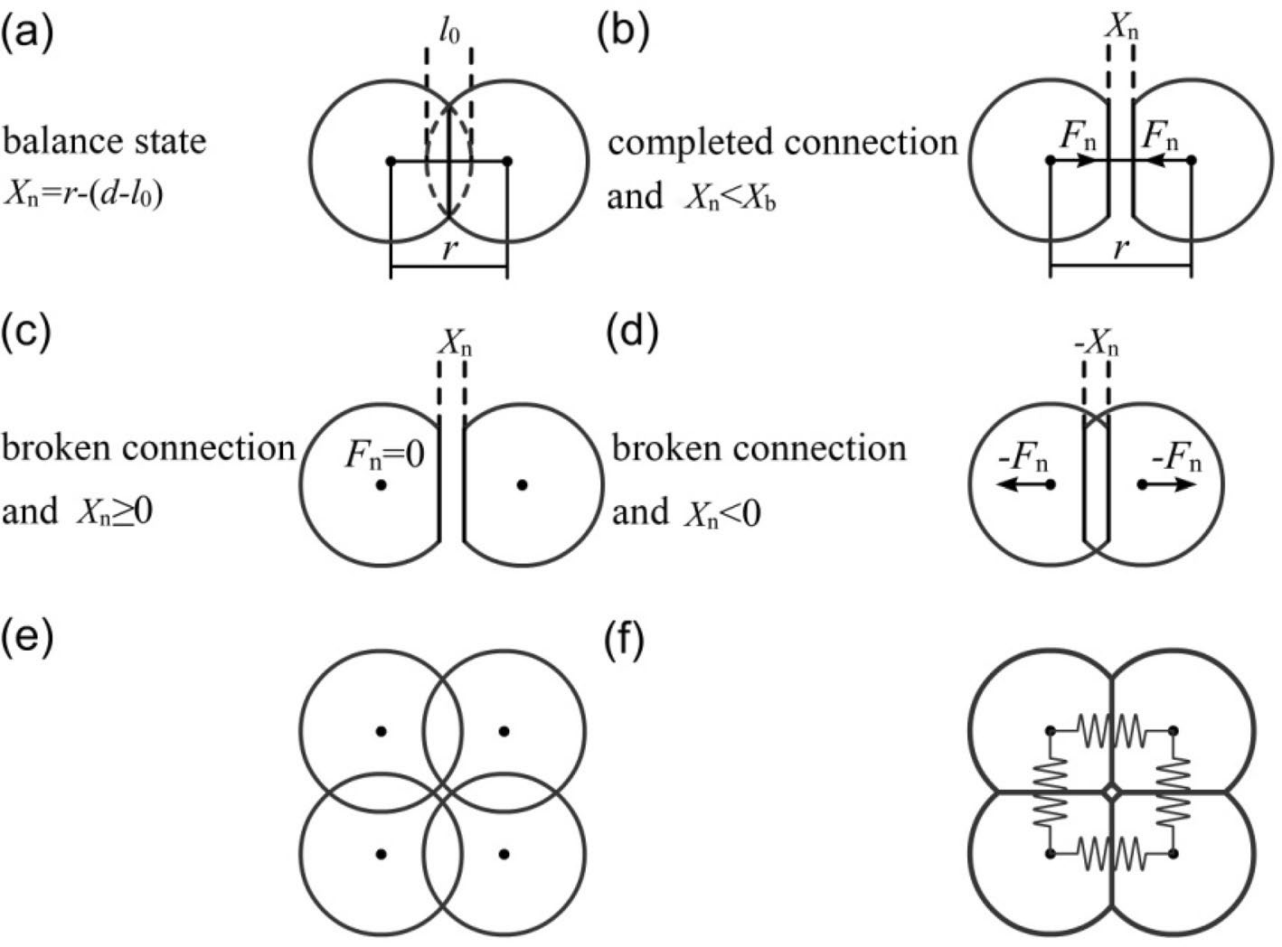
Schematic diagram of the elastic clump model.

As shown in Fig. 2a, the two elements overlap with each other and the normal relative displacement is *l*_0_, the diameter of each element is *d*. According to the Eq. (1), in normal stacking state, the compressive force between the two elements is −*K*_n_*·l*_0_. However, in the proposed elastic clump model, the equilibrium distance is defined as (*d*—*l*_0_), and the relative displacement between the elements can then be calculated by the following equation:

4 $$X_{{\text{n}}} = r - {(}d - l_{{0}} {)}$$

According to this equation, in the state shown in Fig. 2a, the relative displacement *X*_n_ of the two elements is zero and they are in a state of equilibrium. In Fig. 2a, the vertical line segment between two elements is the contact surface of them. The spring forces between the two elements also are determined by the Eq. (1). Such as in Fig. 2b, when the distance between the two elements increases, the *X*_n_ increases and the tensile force between the elements is generated according to the Eq. (1a). The tensile failure criterion of the connection is determined by the Eq. (1). In Fig. 2c, when the relative displacement reaches the breaking displacement *X*_b_, the inter-element connection breaks according to the Eq. (1). However, in order to simulate the unbreakable flexible membrane, the connection between the clump elements will be re-cemented immediately, i.e. the bond between two elements will be restored to an intact status, and therefore the tensile force always exists between them. In Fig. 2d, when two elements are squeezed, repulsive force is generated between them according to the Eq. (1).In the clump model, the shear force is considered, too. The calculation of shear force follows Eq. (2) and Eq. (3), i.e. the equilibrium displacement in the shear direction is zero.

When a connection is declared as a clump connection, the amount of overlap between its two elements will be set as the initial overlap (i.e., the *X*_n_ is 0). When calculating the force between the two elements, the actual overlap between them is subtracted from the initial value. In the traditional clump model^28^, the relative position between clump elements are fixed, and the clump model is almost rigid. However, in the elastic clump model, the relative position is not fixed, so the whole clump is elastic and deformable. As shown in Fig. 2e, the four elements overlap and reach a state of equilibrium (Fig. 2f).

In summary, the only difference between regular elements and clumps is the definition of *X*_n_, i.e. the relative displacement. In clump model, after the equilibrium displacement is set, the calculation of elements forces also follows the equation given in “The contact model of elements” section. Since the re-cementation mechanism is added to the connection of the elements in the clump, the clump is always elastic and deformable, and is difficult to be destroyed.

## The conventional triaxial tests

**Figure 3:**
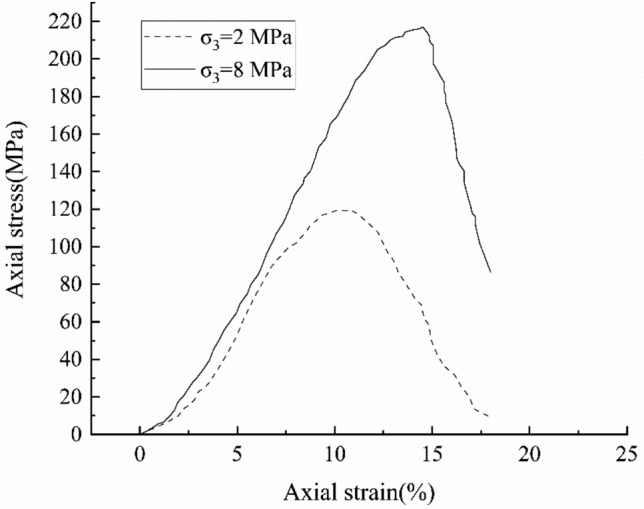
Stress–strain curves of the tests.

The test sample was Jurassic grey fine sandstone of Majiagou, Zigui County, China. The sample was cylindrical with a diameter of 50 mm and a height of 100 mm. Triaxial tests were carried out under the confining pressures of 2 MPa (test 1) and 8 MPa (test 2), respectively. The stress–strain curves are showed in Fig. 3. With the increasing axial strain loading, the stress on the two samples increases first and then decreases when it reaches the peak. The axial strains at the peaks are respectively 10.35% and 14.51% for the test 1 and test 2. At each axial strain loading level, the stress of test 2 (higher confining pressure) is always higher than that of the test 1, because of the high confining pressure, the rock shows greater peak strength. At the initial stage of loading, the stress–strain curve is concave, this is because the micro-cracks in the sample were closed tightly; with the increasing loading, the stress–strain curve increased approximately linearly, which indicates an elastic deformation stage; and then reached the peak strength; after peak strength, the stress decreased rapidly, which indicated the brittle fractures were generated in the rock^25^.

## Numerical simulations of triaxial tests

### The flexible membrane

Based on the elastic clump model, we propose a new flexible membrane principle that allows overlapping among elements. The membrane can prevent elements from leaking out and does not increase the computational complexity. At the same time, due to the overlap of elements, the membrane surface is relatively smooth.

**Figure 4:**
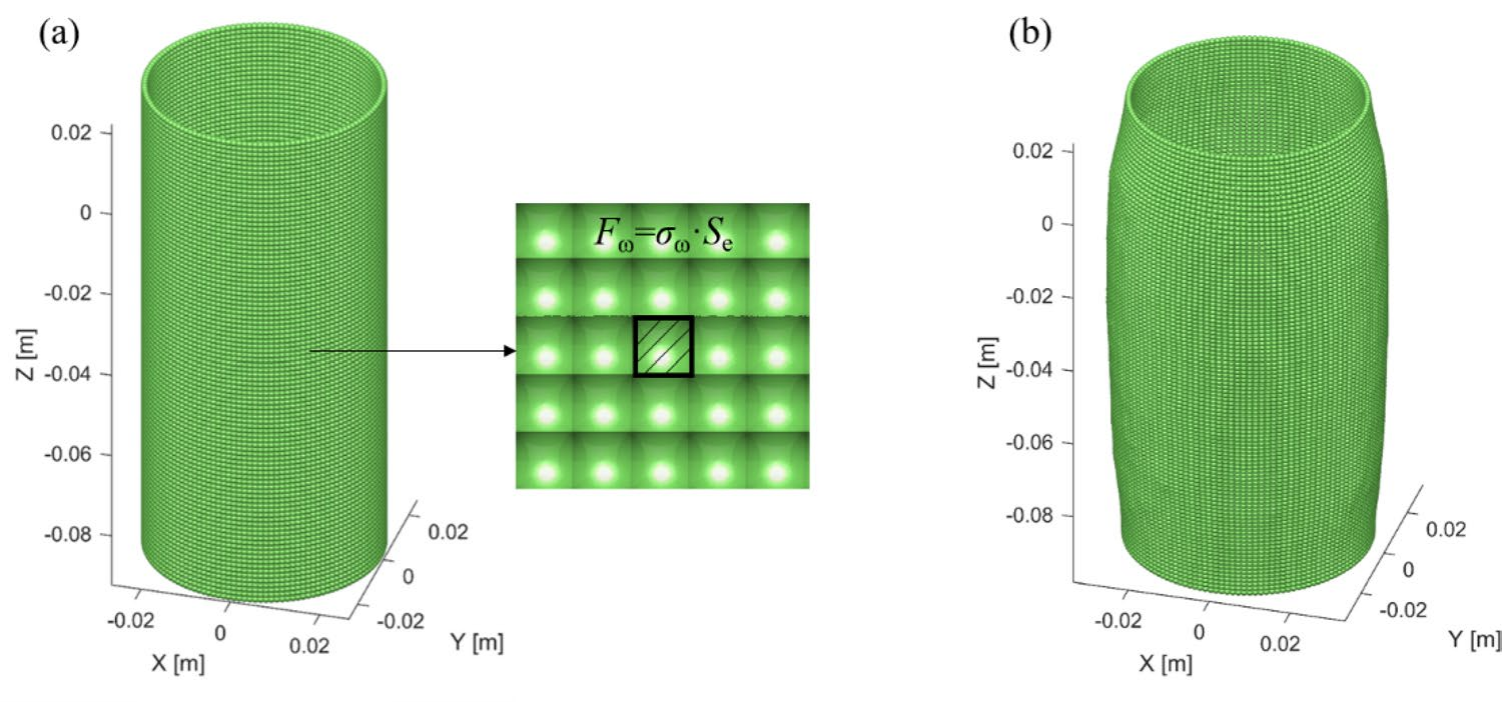
The flexible membrane used in this paper: (**a**) un-deformed membrane with regular packing; (**b**) deformed membrane at 18% axial strain.

As shown in Fig. 4, the flexible membrane is made up of elastic clump model, the elements which made up the clump have the same radius of 0.75 mm and are bonded in a square arrangement, the whole membrane is a clump. As the elements are overlapped with each other, there is no gap between the membrane elements (Fig. 4a), so the membrane surface is relatively smooth compared with normal stacking state. As shown in Fig. 4a, there is no hole on membrane; the membrane elements are assigned the properties of the rubber, and it will not break even if a large deformation occurs, so the sample elements will not escape from the membrane zone. Figure 4b shows the shape of a deformed membrane during numerical simulation, which is expansion deformed but does not rupture.

In real conventional triaxial tests, the confining pressure applied on the membrane is always perpendicular to the surface of the membrane and towards the sample. In order to simulate the effect of the confining pressure, the flexible membrane is decomposed into many quadrilateral regions. As shown in Fig. 4a, the solid line quadrilateral region represents an element, which is surrounded by its four neighboring elements. The confining force (*F*_ω_) on the quadrilateral region is perpendicular to the region surface, and its numerical value equals the product of the area of the solid square(*S*_e_) and the predetermined confining pressure (*σ*_ω_), which can be calculated by:

5 $$F_{\upomega } = \sigma _{\upomega } \cdot S_{e}$$

In each iteration of a numerical simulation, the normal direction of each quadrilateral square is calculated, and the confining force is calculated and applied on the element. The flexible membrane may be deformed during the simulation, however, the confining force will always perpendicular to the membrane surface, so as to simulate the effect of confining pressure in real experiments. There is no cementation between the sample elements and the flexible membrane, only pressure and friction exist, which are calculated by the linear elastic contact theory.

### DEM numerical simulations

The methods introduced above were implemented in the three-dimensional DEM simulation software MatDEM^30,31^. Based on an innovative GPU matrix computing of discrete element method, the software may handle millions of elements in one workstation. It has the advantages of high computational efficiency and powerful secondary development functions, and has been successfully applied in the investigation of landslides^32^, failure of sandstone^6^, tunnel issues^33^, etc. In the study, a three-dimensional discrete element simulator for triaxial tests was developed by using the secondary development functions of MatDEM. The software and the source code of the triaxial tests can be downloaded from http://matdem.com.

**Figure 5:**
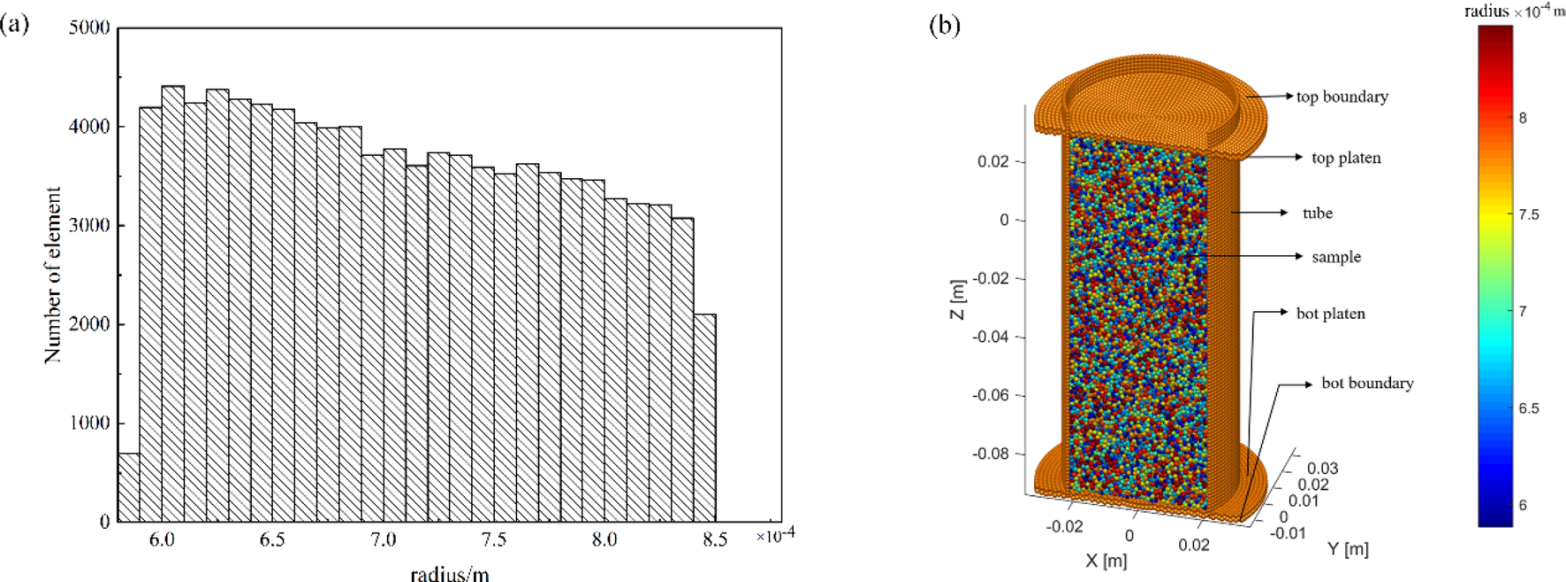
(**a**) The size distribution of elements; (**b**) components of the numerical model.

A numerical model of the triaxial test was established based on the experiments. In the model, the element radius ranges from 0.59 to 0.85 mm, with a mean value of 0.75 mm. Figure 5a shows the detailed element size distribution of the model. As shown in Fig. 5b, the column model, 50 mm in diameter, 100 mm in height, contains approximately 130 thousand elements. In correspondence to the real experiments, the numerical model is consisted of a top boundary, a top pressure plate, a lateral flexible membrane, an internal sample, a bottom pressure plate and a bottom boundary. Pressure plates are also composed of clump elements. The pressure plates and the flexible membrane were set to exceed the sample size, which can prevent the leakage of the sample elements at the junction of the pressure plates and the membrane. The force between the lateral flexible membrane and the pressure plates was neglected in numerical simulations. After the establishment of the model, random initial velocities and the gravity were applied to the sample elements, so as to make a random packing assemblage via the gravity sedimentation.

The mechanical properties of elements are determined by the conversion formulas^30^. In the regular packing model, there are analytical solutions between the micro mechanical parameters of the elements and the macro mechanical properties of the model, i.e. the conversion formulas^31^:

6 $$K_{{\text{n}}} = \frac{\sqrt 2 Ed}{{4(1 - 2\nu )}}$$

7 $$K_{{\text{s}}} = \frac{\sqrt 2 (1 - 5\nu )Ed}{{4(1 + \nu )(1 - 2\nu )}}$$

8 $$X_{{\text{b}}} = \frac{{3K_{{\text{n}}} + K_{{\text{s}}} }}{{6\sqrt 2 K_{{\text{n}}} (K_{{\text{n}}} + K_{{\text{s}}} )}}T{}_{{\text{u}}}d^{2}$$

9 $$F_{{{\text{s}}0}} = \frac{{1 - \sqrt 2 \mu_{{\text{p}}} }}{6}C_{{\text{u}}} d^{2}$$

10 $$\mu_{{\text{p}}} = \frac{ - 2\sqrt 2 + \sqrt 2 I}{{2 + 2I}},\;\;\;\;I = [(1 + \mu_{{\text{i}}}^{2} )^{1/2} + \mu_{{\text{i}}} ]^{2}$$

Five micromechanical parameters of DEM, including normal stiffness (*K*_n_), shear stiffness (*K*_S_), breaking displacement (*X*_b_), initial shear resistance (*F*_s0_) and friction coefficient (*μ*_p_), can be obtained from five macroscopic mechanical properties of materials, including young's modulus (*E*), Poisson's ratio (*ν*), compressive strength (*C*_u_), tensile strength (*T*_u_) and internal friction coefficient (*μ*_i_).

By using the conversion formulas, five mechanical parameters of the elements can be determined by five mechanical properties of rock, including the Young's modulus, Poisson's ratio, tensile strength, compressive strength and internal friction coefficient. Numerical tests indicate that the mechanical properties of the close-packed model determined by the conversion formulas are generally lower than the theoretical values^30,31^, so the input macroscopic mechanical properties of the numerical model are increased appropriately in the modeling. Table 1 shows the mechanical properties of macroscopic model and the mean micro mechanical parameters of model elements. Table 2 shows the mechanical parameters of the flexible membrane.

**Table1:** Macro and micro mechanical properties of the sample.

Macro mechanical properties		Micro mechanical parameters	
Young modulus/GPa	7.00	Normal stiffness/KN m^−1^	4.83 × 10^4^
Poisson ratio	0.22	Shear stiffness/KN m^−1^	1.78 × 10^4^
Compressive strength/MPa	42.00	Breaking displacement/m	1.16 × 10^–6^
Tensile strength/MPa	5.25	Shear resistance/N	1.13 × 10^2^
Friction coefficient	1.96	Element friction coefficient	0.59

**Table2:** Macro and micro mechanical properties of the rubber membrane.

Macro mechanical properties		Micro mechanical parameters	
Young modulus/GPa	7.84 × 10^–3^	Normal stiffness/KN m^−1^	27.78
Poisson ratio	0.10	Shear stiffness/KN·m^−1^	15.44
Compressive strength/MPa	50.00	Breaking displacement/m	2.82 × 10^–7^
Tensile strength/MPa	2.00 × 10^–3^	Shear resistance/N	20.87
Friction coefficient	2.00	Element friction coefficient	0.60

The elements are originally connected to each other to form an intact sample model. In correspondence to the experiments, two sets of numerical experiments were carried out under the confining pressures of 2 MPa and 8 MPa respectively. In the numerical simulations, the bottom boundary is fixed, and the top boundary is moved downward to push the top plate and compress the sample step by step. In order to avoid the strong compressive force between the top plate and the sample, the loading rate must be very small. The total loading displacement of the top boundary was 23.4 mm, the loading process was divided into 600 steps, and the loading rate of the top boundary used in the simulations was 3.9 × 10^–2^ mm/step. Furthermore, a greater average viscosity of elements 0.33 N/m s^−1^ was used to damp the waves in the model, and to avoid buildup of kinetic energy in the isolated system. The time step was 6 × 10^–8^ s. After each loading, the model is run for 130 iterations to damp the generated compressive wave. All calculations were performed on a Tesla V100 GPU Server. The calculation time of a numerical simulation was 2 h.

**Figure 6:**
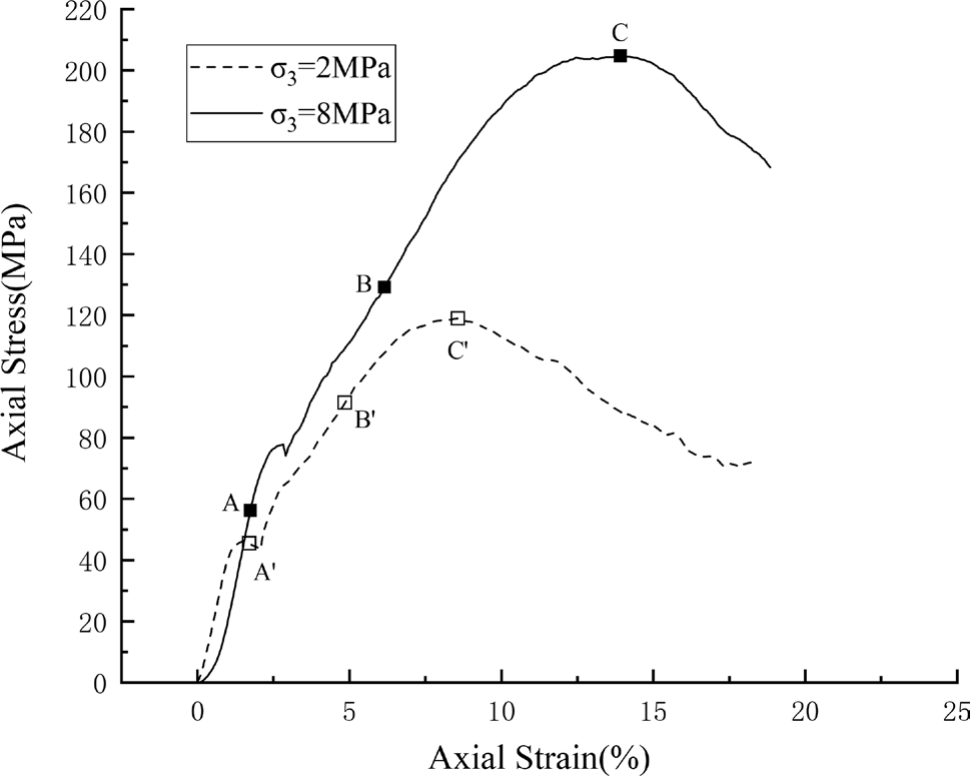
The stress–strain curves of simulations.

Figure 6 shows the results of the DEM simulations under the confining pressures of 2 MPa and 8 MPa, respectively. The axial stress increases with the increasing uniaxial compression and reaches a peak value, after which the stress decreases rapidly, which has a similar trend with the triaxial test curve. The peak values on the stress–strain curves are indicated by the points C and C′. The detailed peak stresses and strains of the DEM simulations and the experimental tests are given in Table 3. In case of both the 2 MPa and 8 MPa confining pressures, the peak stresses and corresponding strains are close to the results of tests. From the above analysis, the results of the DEM simulations and triaxial tests performed highly accordance.

**Table 3:** Comparison of peak stresses and strains between the DEM simulations and the experimental tests.

	Confining pressure/MPa	Peak strain/%	Peak Stress/MPa
Experimental tests	2	10.35	119.16
	8	14.51	216.98
DEM simulations	2	8.52	118.93
	8	13.92	204.75

Note that, the axial stress drops a bit in the early stage of loading. When the confining pressure is 8 MPa, the corresponding strain is 2.4%; when the confining pressure is 2 MPa, the corresponding strain is 1.6%. The drop of stress may be related to the formation of tensile micro fractures, which will be introduced in the “Failure process of the model” section.

## Analysis of numerical simulation results

### Failure process of the model

**Figure 7:**
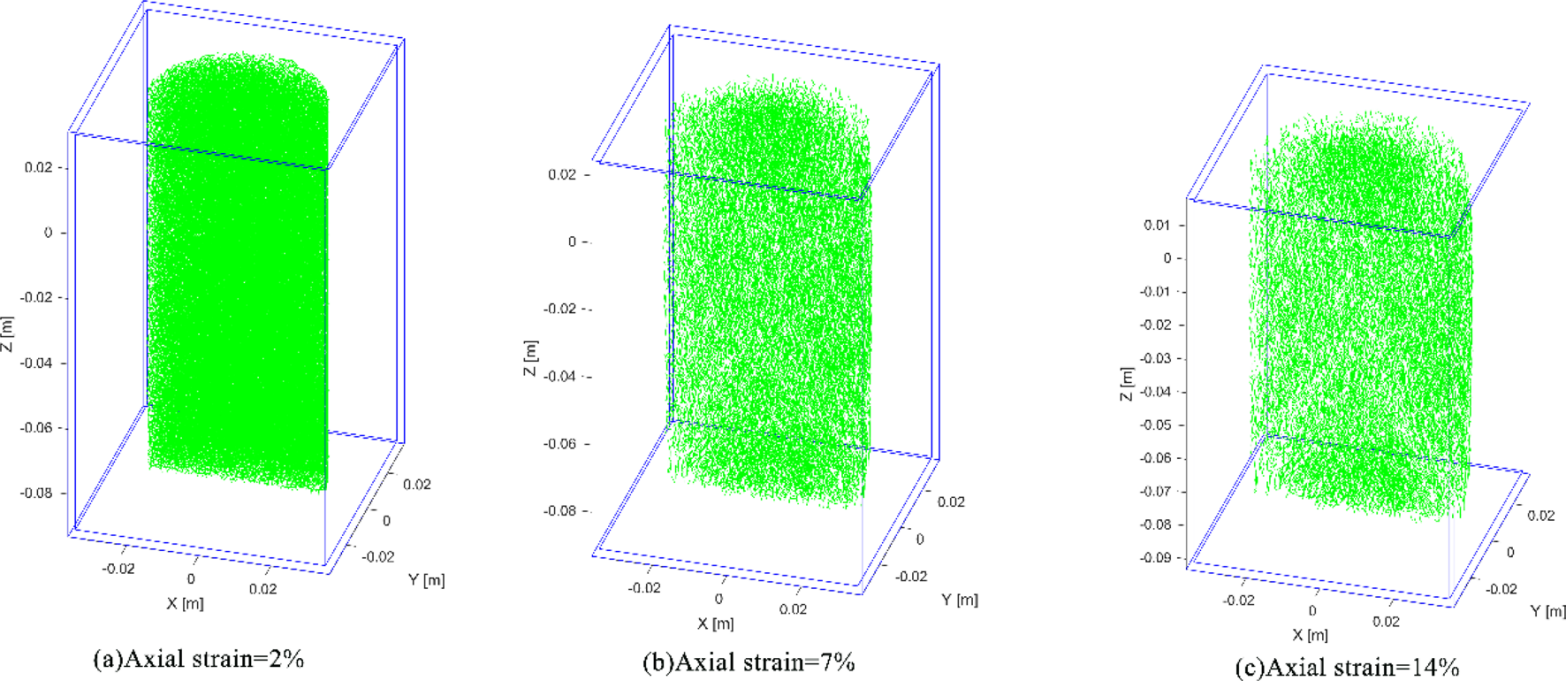
The micro connections of the model under 8 MPa confining pressure (segments indicate intact connections).

**Figure 8:**
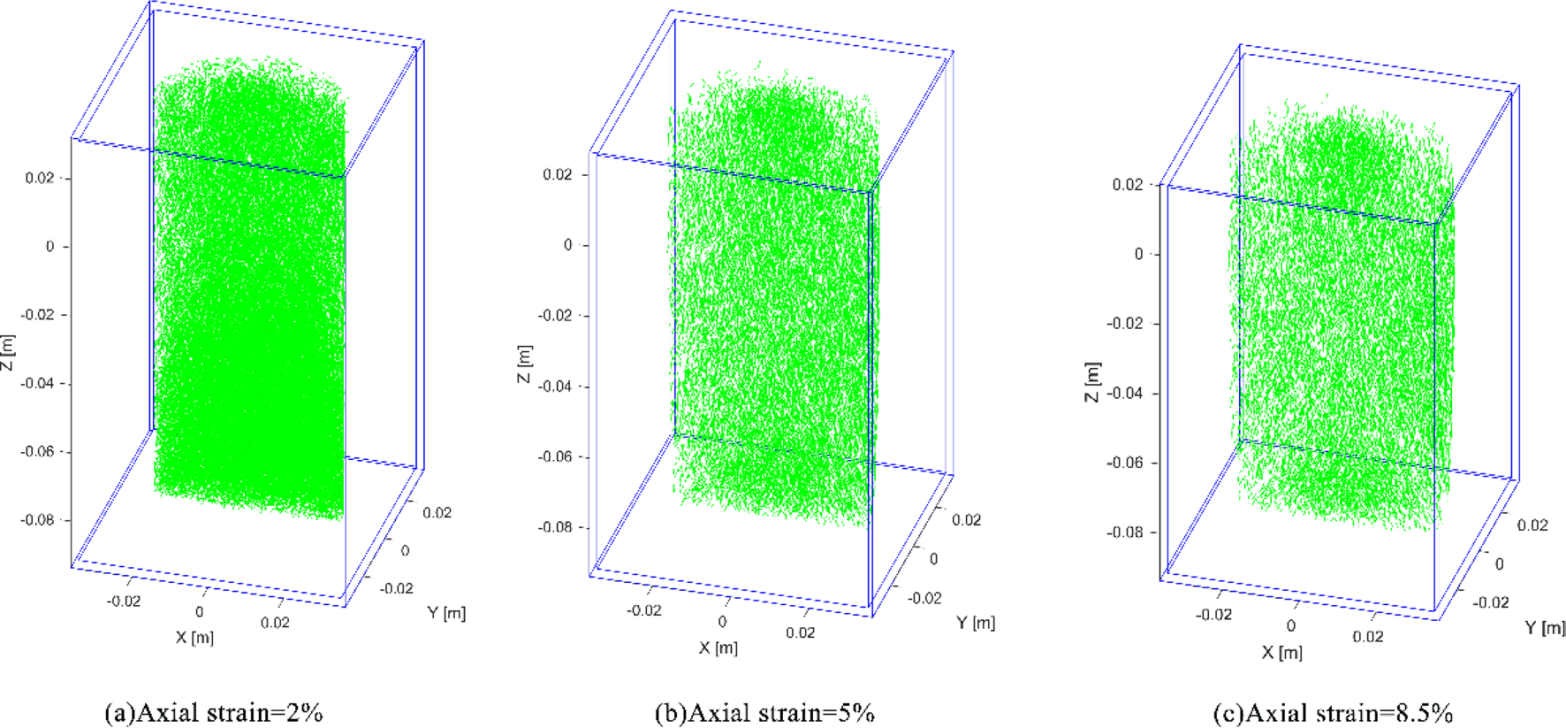
The micro connections of the model under 2 MPa confining pressure (segments indicate intact connections).

Failure processes of the model are associate with the breakage of inter-element connections. The element connection networks of the sample under confining pressure 8 MPa are shown in Fig. 7a-c, which correspond to the points A-C of Fig. 6 and the axial strains of 2%, 7% and 14%, respectively. In the figures, the segments represent intact connections between two elements, and the blank spaces indicate the connections are broken, i.e. micro fractures formed in the rock sample. When the strain is 2%, there is no obvious crack; with the increasing compressive strain, the stress acted on the model increases gradually, producing more micro fractures. When the vertical strain reaches 7% (Fig. 7b), it can be seen that the fracture increases obviously; when the strain is 14%, the model reaches the peak value of stress (Fig. 6), and the X-shape fracture zone is quite clear in Fig. 7c, which is conformed to the conventional rock failure law. The failure process under 2 MPa confining pressure is shown in Fig. 8a-c (correspond to points A’-C’ in Fig. 6), the model shows similar fracture evolution law, and when the peak strain reaches 8.5% (Fig. 8c), X-shape failure zone also generated.

**Figure 9:**
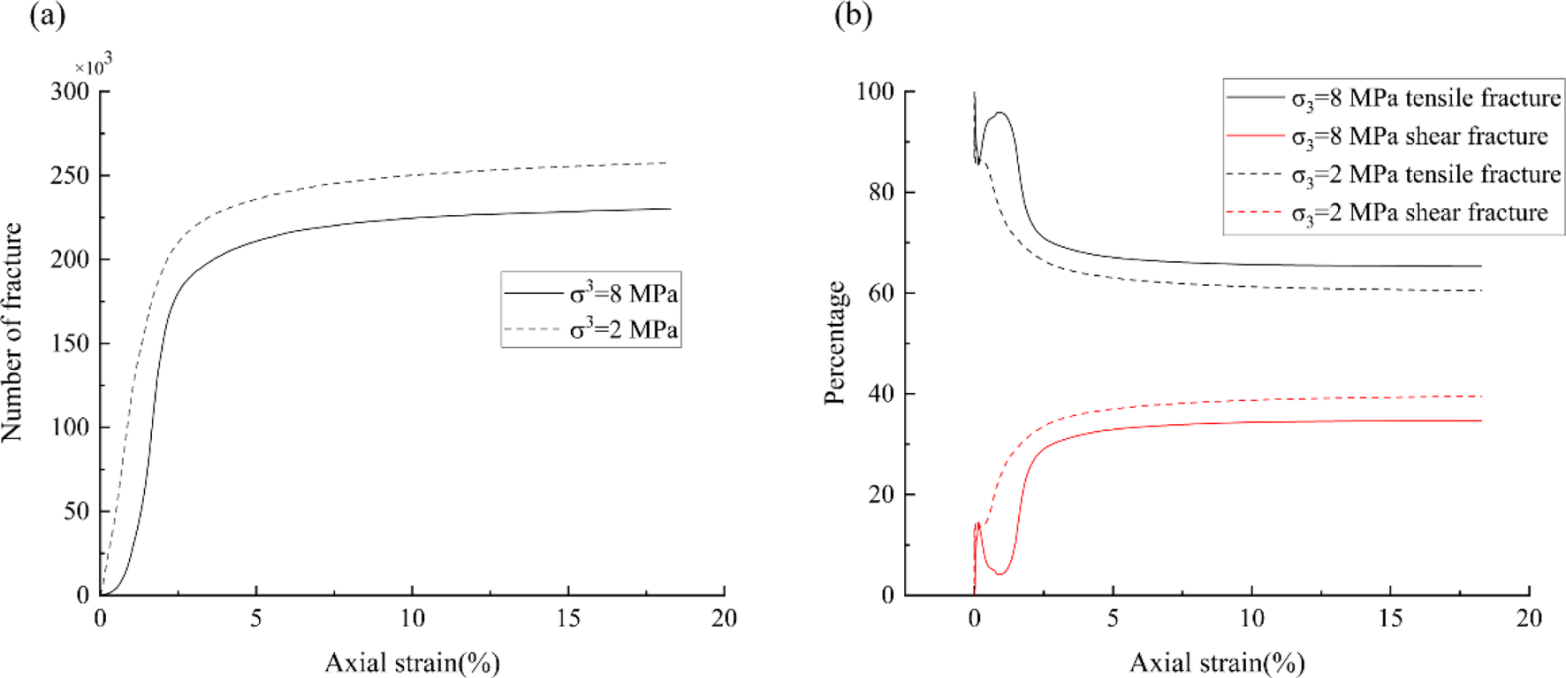
The number of tensile and shear fracture at different confining pressure: (**a**) all fracture; (**b**) percentage of different fracture.

Figure 9a shows the change of the number of fractures in the model under different confining pressures during the loading process. The number of fractures increases with the increasing axial strain, and it increases rapidly before the point of 2.5% strain, after which the growth rate decreases gradually. When the strain reaches about 10%, the number of fractures increases slowly, and the number of fractures tends to be constant. At each strain, the number of fractures under the 2 MPa confining pressure is more than that under the 8 MPa confining pressure. When the confining pressure was increased, less internal fractures were generated, which is consistent with the result of conventional rock tests.

The micro fractures generated during compression can be divided into tensile fractures and shear fractures, and the failure of a rock sample is a result of accumulation of these fractures; microscopic observations of rock in laboratory show that most of the cracks formed in rock compression tests are tensile fractures, and shear fractures can also form, but they usually do not propagate in their own plane, the ultimate failure of the sample must be achieved by connecting tensile fractures to form macro shear fractures^34,35^. Figure 9b shows the percentage change of the tensile fractures and shear fractures during the compressions. The overall trend of the percentage of the tensile fractures is decreasing. However, in the early stage of the compression, there is an obvious increase in the percentage of tensile fractures. Particularly, the percentage of tensile fractures increase dramatically to approximately 97% in case of the 8 MPa confining. In both two confining pressures, the stresses drop (Fig. 6) after the increase of tensile fracture percentage. Therefore, it is inferred that the drop of the stress in the early stage of compression is due to the fast increase of tensile fractures, which also was observed in compressive tests of close-packed discrete element models^30^.

**Figure 10:**
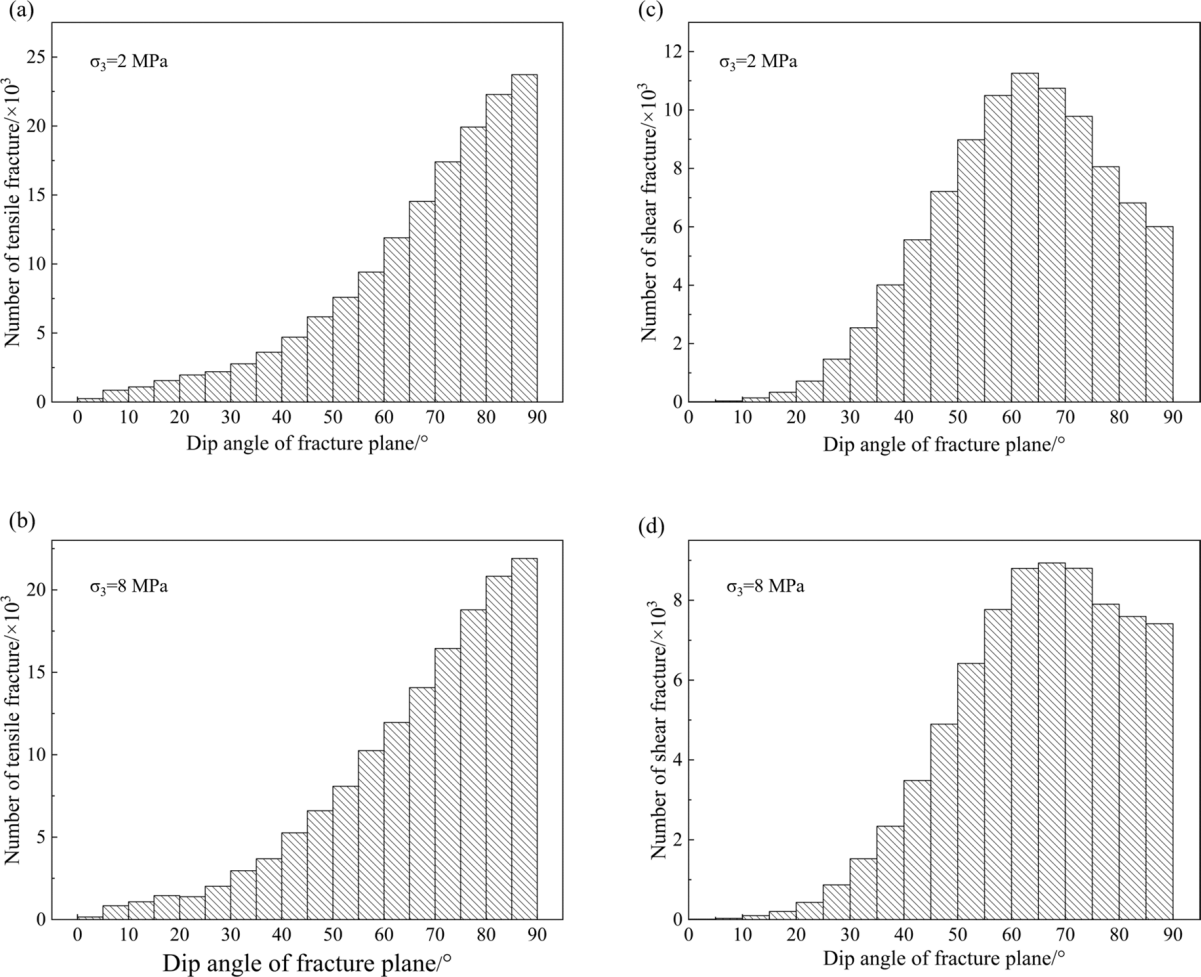
Number of fractures versus dip angle under different confining pressures: (**a**, **b**) tensile fractures; (**c**, **d**) shear fractures.

For a broken connection of two elements, the fracture plane is defined as a small disc located between the two elements and perpendicular to the connection. The dip angle (the angle between structural plane and horizontal plane) distributions of fracture planes at peak strengths are illustrated in Fig. 10. As shown in Fig. 10a, b, 72.3% of the tensile fractures’ dip angle are greater than 60° under 2 MPa confining pressure, and 63.8% are greater than 60° under 8 MPa confining pressure. Therefore, most of the tensile fractures are approximately parallel to the maximum principal stress, and it tends to form in the direction parallel to the maximum principal stress. The dip angle distribution of shear fractures is quite different. In Fig. 10c,d, the dip angles of most fracture planes are between 50° and 80°, and there is an obvious quantity peak at about 65°, which is very close to the preset internal friction angle of 63° (tan (63°) is 1.96, see the friction coefficient in Table 1).

### Movement law of elements

**Figure 11:**
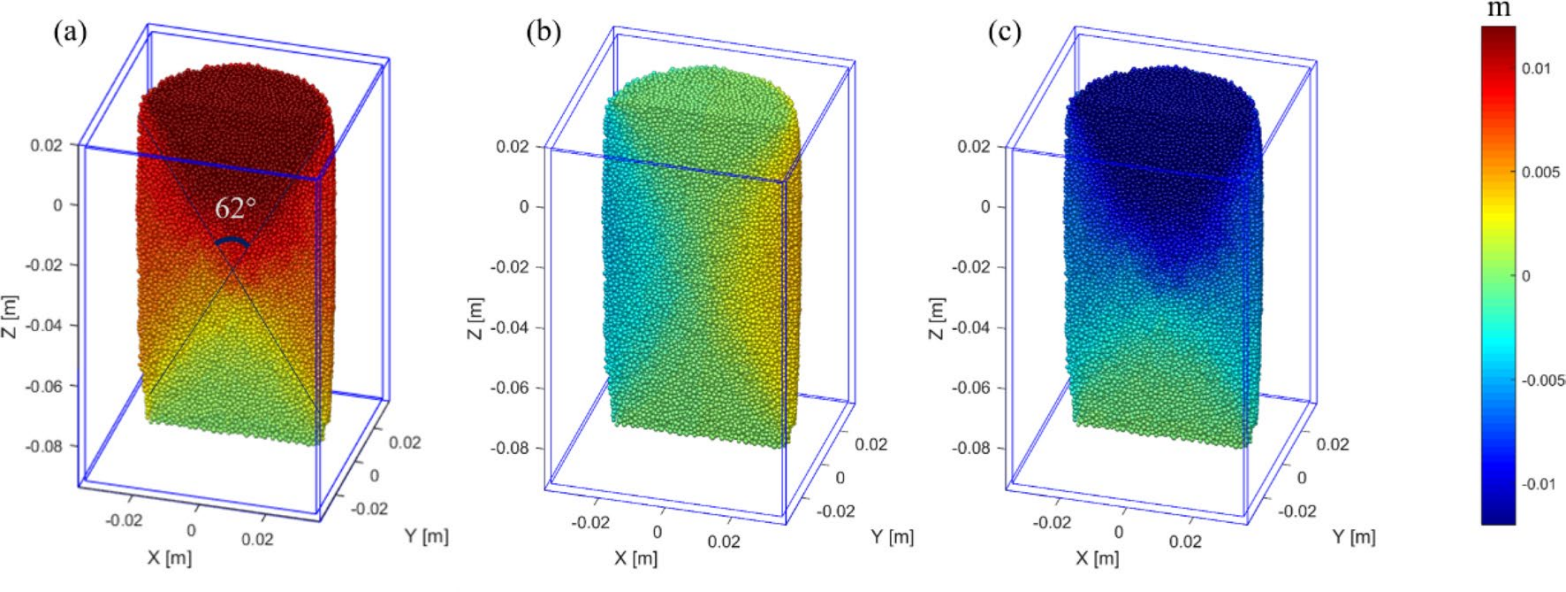
The displacement field at strain 8.5% under 2 MPa confining pressure: (**a**) total displacement; (**b**) X-displacement; (**c**) Z-displacement.

The element displacement field at the strain 8.5% under the 2 MPa confining pressure is shown in Fig. 11. In the figure, when the axial strain reaches the peak strain, the displacement field of the model can be clearly divided into three regions: I, II and III. The region I is located in the upper part of the model and has an inverted cone shape. This region has almost the same total displacement (Fig. 11a) and the z-component (Fig. 11c), which indicates these elements are mainly moved downward and dilated a bit during the compression. The region III is located at the bottom side of the model, and its shape was similar to region I, but there is almost no displacement. The region II is located on the lateral sides of the model, the total displacement of this region is between that of the region I and region III, and the x-component of the elements shows a lateral expansion (Fig. 11, region II). Due to different displacement fields, obvious interface between the three regions can be observed. It is estimated that the intersection angle of the two shear fracture planes is about 62° (Fig. 11a), which is very close to the input internal friction angle of 63°, and shows that the formation of the shear fracture in the rock is in accordance with the Mohr–Coulomb criterion. The displacement zoning characteristics of the three regions correspond to the rock deformation failure mode: axial compression, lateral expansion and shear failure.

**Figure 12:**
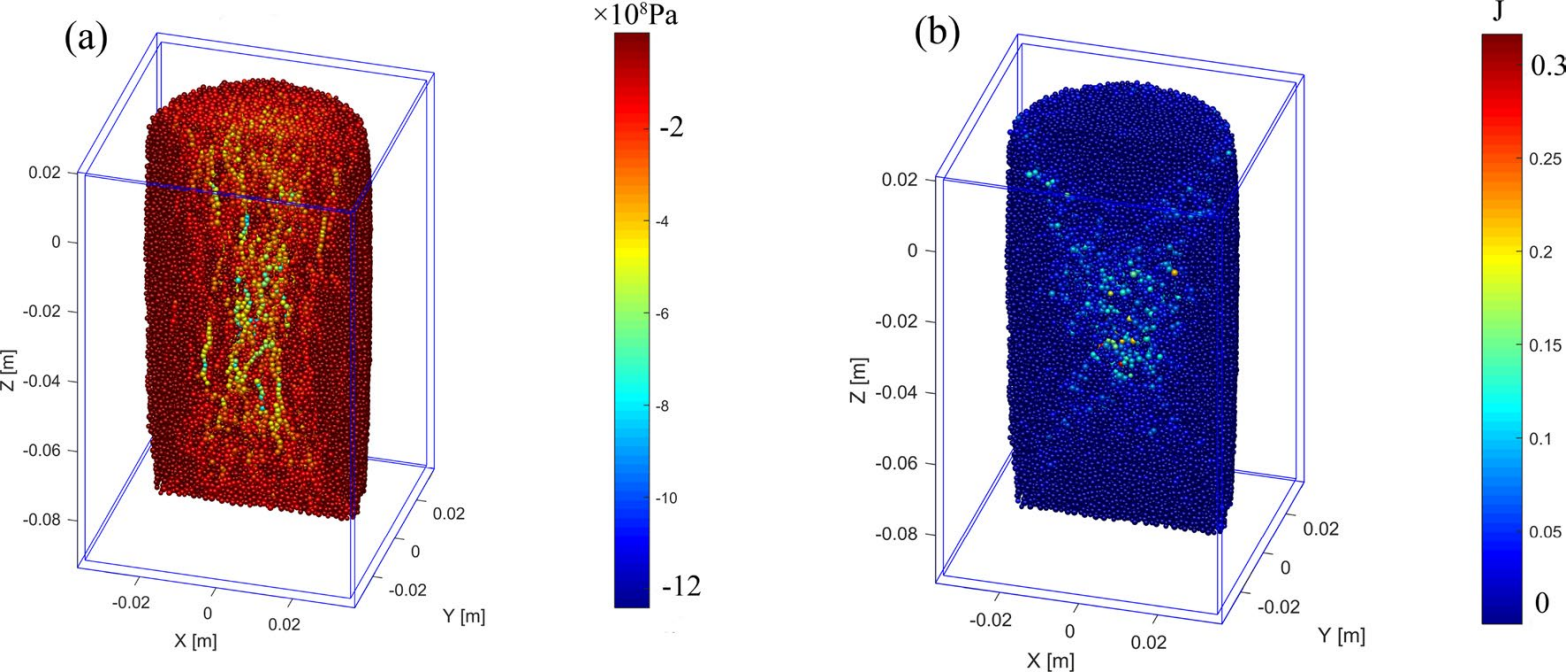
The stress field and slipping frictional heat field at strain 8.5% under 2 MPa confining pressure: (**a**) stress field; (**b**) slipping frictional heat field.

The Fig. 12a shows the stress distribution in the model under the confining pressure of 2 MPa. Due to the compression, the force chains can be observed along the direction of compression. The stress gradually concentrated in the middle part of the model, forming a high stress area. When the compressive strain is 8.5% (Fig. 12a), the region of force chains shows a funnel shape, which is similar to that of the Fig. 11b.

With the loading, the X-shaped shear failure is formed gradually, and the elements slip relatively along the failure surface. Figure 12b shows the slipping frictional heat field in the model at 8.5% strain under the confining pressure of 2 MPa. It can be observed that the slipping frictional heat is concentrated on the failure surface, which also shows an obvious X-shaped distribution.

### Energy conversion in the model

**Figure 13:**
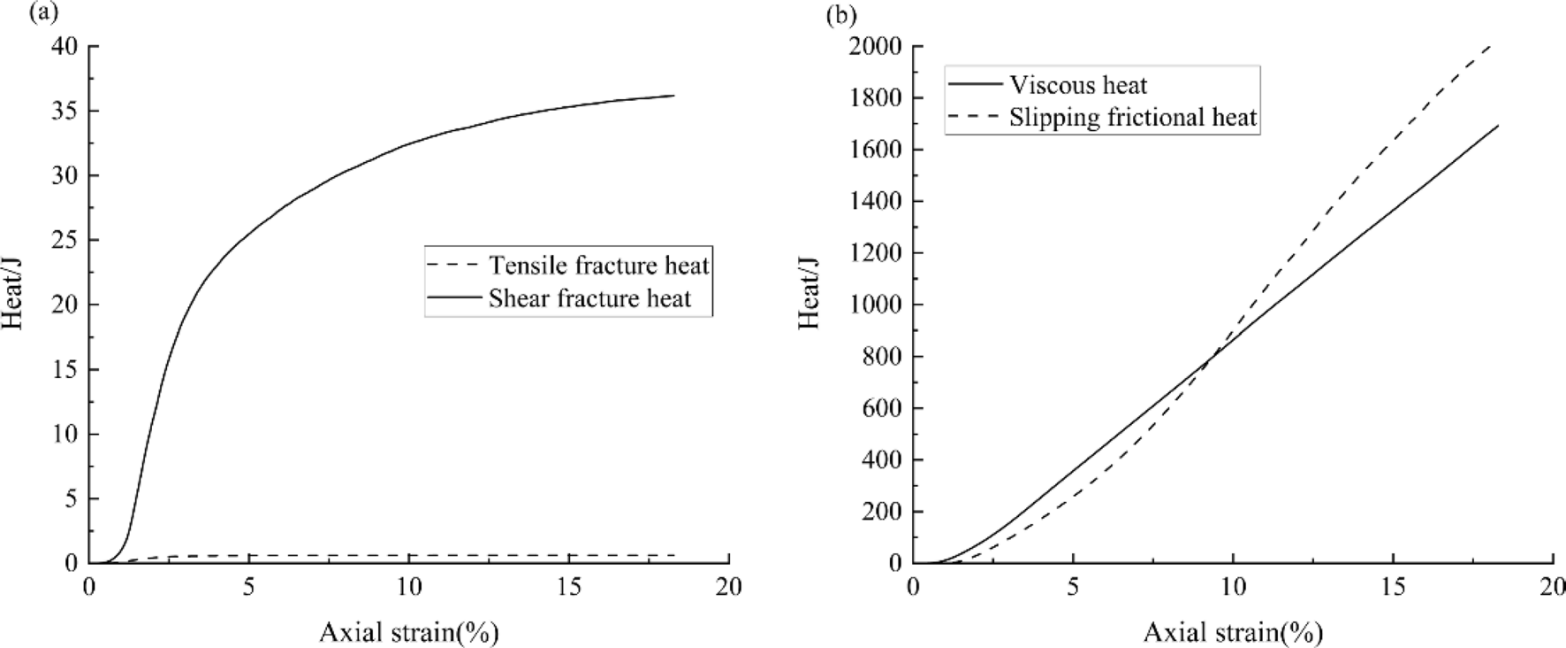
Heat in the model during loading under the 2 MPa confining pressure: (**a**) tensile fracture heat and shear fracture heat; (**b**) viscous heat and slipping frictional heat.

In the numerical simulation, the mechanical energy of the sample increases with the increasing work of external compressive forces. The energy is stored in the model in the form of the elastic potential energy, or is converted to viscous heat, frictional heat and fracture heat due to the effect of viscous force, frictional force and fracturing, respectively^30^. The fracture heat can be divided into the normal fracture heat, shear fracture heat that generated during the failure of normal springs and shear springs. Figure 13a shows the curves of the normal fracture and shear fracture heat generation during the loading process (the confining pressure is 2 MPa). Actually, the compression and failure process of a sample is the process of accumulation and release of elastic potential energy and the generation of heat. During the compression, the absolute value and relative growth rate of shear fracture heat are much higher than that of tensile fracture heat, indicating that shear fracture plays a leading role in energy consumption during the formation of micro fractures, although the number of shear fracture is less. Note that the tensile fracture heat increases at the beginning of compression, and almost maintains constant after the 2.5% compressive strain. The average shear fracture heat of a bond is 3.56 × 10^-4^ J and 1.3 × 10^-3^ J under the confining pressures of 2 MPa and 8 MPa, respectively. Therefore, the average shear fracture heat of a bond increases with the increasing confining pressure, and fractures are more likely to generate under low confining pressures.

Most of the heat in the model is dissipated in the form of viscous heat and slipping heat, and the viscous heat is produced by viscosity which is set to eliminate stress waves. Figure 13b shows the curves of the viscous and slipping heat generation. The viscous heat increases almost linearly, because it was generated due to the effect of the viscous force, which is used to eliminate compressive wave during each compressive step. The frictional heat is generated when two elements sliding toward each other. So, it is related to the number of fractures and also the stresses. As shown in Fig. 13b, the frictional heat is relative less than the viscous heat at the initial stage, but increases rapidly with the increasing compressive strain. The sample failures and the compressive stress drops when the strain exceeds 8% (see Fig. 6), after that, the frictional heat increases almost linearly due to the continuous sliding along the X-shape shear zone.

## Conclusions

This paper proposed a 3D discrete element model based on a new elastic model and a flexible membrane for the simulation of the conversional triaxial tests. The flexible membrane composed of overlapping elastic clump elements has the three advantages: (1) it is completely elastic and will not break; (2) there is no hole on the membrane surface, so it can prevent the sample from leaking out, and the surface of the membrane is smooth; (3) it can apply the confining pressure accurately and conveniently.

In the numerical triaxial tests, with the increasing compression, the sample was broken into several parts, and the stress status varied. The displacement field in the model can be divided into three parts: the upper part, the lateral part and the lower part. Under the loading, the displacement law shows compression in the upper part, static in the lower part, expansion in the side, and finally X-shape interface of displacement can be observed. The dip angle of most shear fractures is close to the internal friction angle of the model, which indicates that the micro shear failure of the rock also follows the Mohr–Coulomb criterion. And tensile fractures tend to be parallel to the direction of the maximum principal stress and have an important influence on the occurrence of peak strength. Energy analysis indicates that shear fractures consume much more energy than tensile fractures, so most of the micro fractures in the model are tensile fractures. In the early stage of loading, the energy in the model is dissipated mainly in the form of viscous heat, when it reaches the peak strength, the elements slipped along the X-shape shear failure and frictional heat generated.

By using the flexible membrane, the conversional triaxial tests were well simulated. It was able to decipher the failure process of rock sample based on the comprehensive analysis of the distribution and variation of stresses, displacements, energy and fractures of the model. Further, the flexible membrane and the new model can be widely used in studying the micro mechanism of rock failure processes.
